# INCLUDING FAMILY CAREGIVERS IN SERIOUSLY ILL VETERANS' CARE: A MIXED-METHODS STUDY

**DOI:** 10.1093/geroni/igz038.751

**Published:** 2019-11-08

**Authors:** Nina R Sperber, Nathan A Boucher, Roxana Delgado, Megan Shepherd-Banigan, Kevin McKenna, Madison Moore, Margaret Kabat, Courtney H Van Houtven

**Affiliations:** 1 Duke University, DURHAM, North Carolina, United States; 2 University of Texas Health San Antonio, San Antonio, Texas, United States; 3 The Elizabeth Dole Foundation, Washington, District of Columbia, United States; 4 Department of Veterans Affairs, Washington, District of Columbia, United States

## Abstract

Family caregivers often serve as unpaid members of the home- and community-based care workforce for individuals with serious illness; as key partners in the home-clinic continuum, they should be included in healthcare teams. Without formal recognition, inconsistent identification of caregivers will remain, engendering communication gaps between caregivers and providers and difficulty connecting with supportive services. The Campaign for Inclusive Care is an initiative within the Veterans Administration Health Care System to improve practices for including caregivers in treatment planning and decisions. We define inclusive care using literature review, provider interviews, and a caregiver survey. We found that inclusive care involves clear definition of caregiver role, system policies for inclusion, assessment of caregiver capacity, explicit involvement of caregivers, and mutuality in caregiver/provider communication. We recommend solutions based on the definition, informative for development of a national caregiver strategy, required by the 2018 Recognize, Assist, Include, Support, and Engage Family Caregivers Act.

